# Long-Term Prognostic Impact of Right Ventricular Dysfunction in Patients with COVID-19

**DOI:** 10.3390/jpm12020162

**Published:** 2022-01-26

**Authors:** Fernando Scudiero, Angelo Silverio, Iacopo Muraca, Vincenzo Russo, Marco Di Maio, Antonio Silvestro, Davide Personeni, Rodolfo Citro, Mario Enrico Canonico, Gennaro Galasso, Italo Porto, Guido Parodi

**Affiliations:** 1 Division of Cardiology, “Bolognini” Hospital, ASST Bergamo est, 24068 Seriate, Italy; fernandoscudiero@gmail.com (F.S.); antoniosilvestro@libero.it (A.S.); davide.personeni@asst-bergamoest.it (D.P.); 2 Department of Medicine, Surgery and Dentistry, University of Salerno, 84081 Baronissi, Italy; marcodimaio88@gmail.com (M.D.M.); rodolfocitro@gmail.com (R.C.); ggalasso@unisa.it (G.G.); 3 Division of Interventional Cardiology, Cardiothoracovascular Department, Careggi University Hospital, 50141 Florence, Italy; iacopo.muraca@gmail.com; 4 Division of Cardiology Department of Translational Medical Sciences, University of Campania “Luigi Vanvitelli”—Monaldi and Cotugno Hospital, 81100 Naples, Italy; v.p.russo@libero.it; 5 Cardiology Clinic, Sassari University Hospital, 07100 Sassari, Italy; mecanonico@me.com (M.E.C.); parodiguido@gmail.com (G.P.); 6 Department of Internal Medicine and Medical Specialties (DIMI), Clinic of Cardiovascular Diseases, University of Genoa, 16132 Genoa, Italy; italo.porto@unige.it

**Keywords:** COVID-19, right ventricular dysfunction, long-term outcome

## Abstract

The characteristics and clinical course of hospitalized patients with coronavirus disease 2019 (COVID-19) have been widely described, while long-term data are still poor. The aim of this study was to evaluate the long-term clinical outcome and its association with right ventricular (RV) dysfunction in hospitalized patients with COVID-19. This was a prospective multicenter study of consecutive COVID-19 patients hospitalized at seven Italian Hospitals from 28 February to 20 April 2020. The study population was divided into two groups according to echocardiographic evidence of RV dysfunction. The primary study outcome was 1-year mortality. The propensity score matching was performed to balance for potential baseline confounders. The study population consisted of 224 patients (mean age 69 ± 14, male sex 62%); RV dysfunction was diagnosed in 63 cases (28%). Patients with RV dysfunction were older (75 vs. 67 years, *p* < 0.001), had higher prevenance of coronary artery disease (27% vs. 11%, *p* = 0.003), and lower left ventricular ejection fraction (50% vs. 55%, *p* < 0.001). The rate of 1-year mortality (67% vs. 28%; *p* ≤ 0.001) was significantly higher in patients with RV dysfunction compared with patients without. After propensity score matching, patients with RV dysfunction showed a worse long-term survival (62% vs. 29%, *p* < 0.001). The multivariable Cox regression model showed an independent association of RV dysfunction with 1-year mortality. RV dysfunction is a relatively common finding in hospitalized COVID-19 patients, and it is independently associated with an increased risk of 1-year mortality.

## 1. Introduction

Severe acute respiratory syndrome coronavirus 2 (SARS-CoV-2) is a highly pathogenic human coronavirus recognized as the cause of the coronavirus disease 2019 (COVID-19). The outbreak sparked in Wuhan, capital city of Hubei province in China, and spread rapidly to other countries, reaching devastating pandemic proportion [1]. The fast-growing understanding of clinical features of COVID-19 demonstrated a high risk of life-threatening conditions (e.g., sepsis, respiratory failure, heart failure, acute pulmonary embolisms etc.) during the acute phase of illness [2]; several studies have been conducted to evaluate the clinical features associated with the risk of life-threatening complications and mortality during the hospitalization [3]. Among comorbidities, cardiovascular diseases (CVD) are very common in COVID-19 patients and have been associated with higher risk of in-hospital mortality [4]. Indeed, patients with COVID-19 can experience cardiovascular involvement including arrhythmias, acute coronary syndromes, cardiogenic shock, myocarditis, and pulmonary embolism [5,6,7,8,9,10,11]. Previous studies described the association between right ventricular (RV) dysfunction and early mortality, which was probably due to the close relationship between heart and lung function [12,13,14,15,16]. However, the impact of RV dysfunction on the long-term impact is currently unknown.

The aim of this multicenter study was to evaluate the long-term clinical outcome and to assess the association between RV dysfunction and one-year mortality in a multicenter, registry-based cohort of hospitalized patients with COVID-19.

## 2. Methods

### 2.1. Study Design

This was a multicenter prospective observational study including patients with confirmed diagnosis of COVID-19 admitted at 7 hospitals throughout the Italian Country (Bergamo, Naples, Sassari, and Salerno provinces) from 28 February to 20 April 2020. COVID-19 diagnosis was initially based on the World Health Organization criteria, and all cases were confirmed by real-time reverse transcriptase-polymerase chain reaction analysis of throat swab specimens [17].

All patients included in this study were evaluated by the hospital cardiology service and underwent transthoracic echocardiography (TTE) within 48 h from admission. To minimize the exposure to COVID-19, each referral for TTE was confirmed as appropriate by one consultant cardiologist [12,18]. Echocardiographic data were retrospectively analyzed. This study was conducted according to the Declaration of Helsinki and approved by the institutional ethics committees. The requirement for informed consent from individual patients was waived due to the observational design of the study.

### 2.2. Study Measures

The baseline demographic, clinical, laboratory, and TTE data were collected and recorded on an electronic datasheet. In all patients, demographic (age, gender, height, and weight), clinical (comorbidities, pharmacological therapy before and during hospitalization), laboratory (D-dimer, N-terminal pro-brain natriuretic peptide, and high-sensitivity troponin), and echocardiographic data were collected.

Information on patient clinical course (admission in intensive care unit and respiratory support measures) and in-hospital complications were systematically recorded. Acute respiratory distress syndrome (ARDS) diagnosis was defined according to the Berlin definition [19]. Acute myocardial injury was diagnosed in patients with elevated cardiac troponin levels with at least one value above the 99th percentile upper reference limit [20].

### 2.3. Transthoracic Echocardiography

TTE was performed in accordance with the current guidelines [21,22]. Echocardiographic analysis included the evaluation of left ventricular end-diastolic (LVEDV) and end-systolic volumes (LVESV). Left ventricular systolic function was assessed by determining the left ventricular ejection fraction (LVEF) through biplane analysis using the modified Simpson’s rule. Left ventricular systolic function was assessed by determining left ventricular ejection fraction (LVEF) through biplane analysis using the modified Simpson’s rule. As a parameter of global right ventricular (RV) function, tricuspid annular plane systolic excursion (TAPSE), which reflects the base to apex shortening of the right ventricle in systole, was assessed. After adjusting the echo transducer at the level of the RV chamber to achieve optimal visualization of the RV, TAPSE was obtained by aligning the M-mode linear cursor to the lateral tricuspid annulus and calculated as previously described. RV dysfunction was defined by TAPSE value < 17 mm in accordance with the current guidelines [22]. Systolic pulmonary artery pressure (sPAP) was derived from the tricuspid regurgitant jet velocity using systolic trans-tricuspid pressure gradient calculated by the modified Bernoulli equation and the addition of estimated right atrial pressure according to inferior vena cava dimension and inspiratory distensibility [22]. Pulmonary hypertension based on echocardiographic findings are defined according to European Society of Cardiology (ESC) guidelines: a tricuspid regurgitation velocity >2.8 to 2.9 m/s, corresponding to an SPAP of approximately 36 mm Hg, assuming a right atrium pressure of 3 to 5 mm Hg, indicates elevated PA pressure [23].

### 2.4. Follow-Up and Study Endpoint

All recovered patients had scheduled telephonic follow-up at 6 and 12 months from discharge. All other possible information gathered from hospital readmission charts or by referring physicians, relatives, or municipality vital registries, were prospectively entered into an electronical database.

The primary endpoint of this study was to evaluate the association between the RV dysfunction and 1-year mortality.

### 2.5. Statistical Analysis

Distribution of continuous data was assessed with the Kolmogorov–Smirnov test. Normally distributed variables were expressed as mean ± standard deviation, whereas non-normal distributed ones were expressed as median and interquartile range. Categorical variables were reported as numbers and percentages. Continuous normally distributed variables were compared by using Student’s *t*-test; differences between non-normally distributed variables were tested with the Mann-Whitney U test. Categorical variables were compared with chi-squared test, or Fisher exact test, when appropriate. Survival curves were generated by using the Kaplan–Meier method, and differences among groups were investigated with the Log-Rank test.

Due to differences in baseline characteristics between patients with vs. those without RV dysfunction, a propensity score-matched analysis (1:1) was conducted to obtain a covariate-balanced control group. Covariates included in the model were those that were significantly different between the two study groups (age, coronary artery disease, heart failure, LVEF, chronic kidney disease, chronic obstructive pulmonary disease). We performed nearest neighborhood matching with Mahalanobis distance (0.25-SD distance tolerance caliper). Bias reduction was assessed by comparing the standardized difference for propensity score and the other covariates before and after matching between the two groups (a value <10% after matching indicates inconsequential imbalance).

The risk of 1-year mortality in patients with vs. those without RV dysfunction was calculated using the Cox proportional hazard regression model and presented as unadjusted and adjusted hazard ratios (HR) with 95% confidence intervals.

The proportional hazards assumption was assessed and satisfied graphically by plotting log (−log) survival curves against log survival time for each predictor category and verifying whether curves were parallel.

To account for potential confounders related to patients’ baseline clinical profile and the severity of in-hospital course, we performed a multivariable analysis. We used a parsimonious model including variables with *p* < 0.10 by the univariate test as a candidate for the multivariate analysis. The risk of overfitting was controlled by using a ratio of at least 1:10 for the number of explanatory variables and sample size. Model discrimination was assessed with the C statistic and goodness of fit with the Hosmer–Lemeshow test. Multicollinearity was assessed using collinearity diagnostics; the variance inflation factors showed no significant collinearity (<2.5) among the covariates.

A *p*-value < 0.05 was considered significant. All tests were two-sided. Analyses were performed with SPSS statistical package, Version 21 (IBM Corp., Armonk, NY, USA) and R version 3.5.1 (R Foundation for Statistical Computing, Vienna, Austria).

## 3. Results

### 3.1. Study Population

During the study period, 224 consecutive COVID-19 patients were enrolled and divided into two groups according to the TTE evidence of RV dysfunction. Normal RV function was detected in 161 (72%) patients and RV dysfunction was detected in 63 (28%) patients. The baseline demographic and clinical features of the two study groups and of the propensity-matched cohort are summarized in Table 1a. In the overall study population, patients with RV dysfunction were older (75 ± 11 vs. 67 ± 14 years, *p* < 0.001), had higher prevalence of coronary artery disease (27% vs. 11%; *p* = 0.003), heart failure (22% vs. 5%; *p* < 0.001), chronic obstructive pulmonary disease (38% vs. 13%; *p* < 0.001), and chronic kidney disease (39% vs. 12%; *p* < 0.001). No differences in terms of COVID-19 in-hospital medications were reported.

### 3.2. Echocardiography

Left ventricular ejection fraction (LVEF) was lower in patients with RV dysfunction (55% (53–60) vs. 50% (43–55); *p* < 0.001), while no statistical differences regarding the LVESV and LVEDV were observed (Table 1b). Of course, patients with RV dysfunction showed lower TAPSE values; they also showed higher incidence of pulmonary hypertension as compared with those without RV dysfunction (85% vs. 32%; *p* < 0.001).

### 3.3. RV Dysfunction and Outcome

Only one patient was lost at follow-up (follow-up completion > 99%). The proportions of adverse events during the hospitalization are summarized in Table 2.

**Figure 1:**
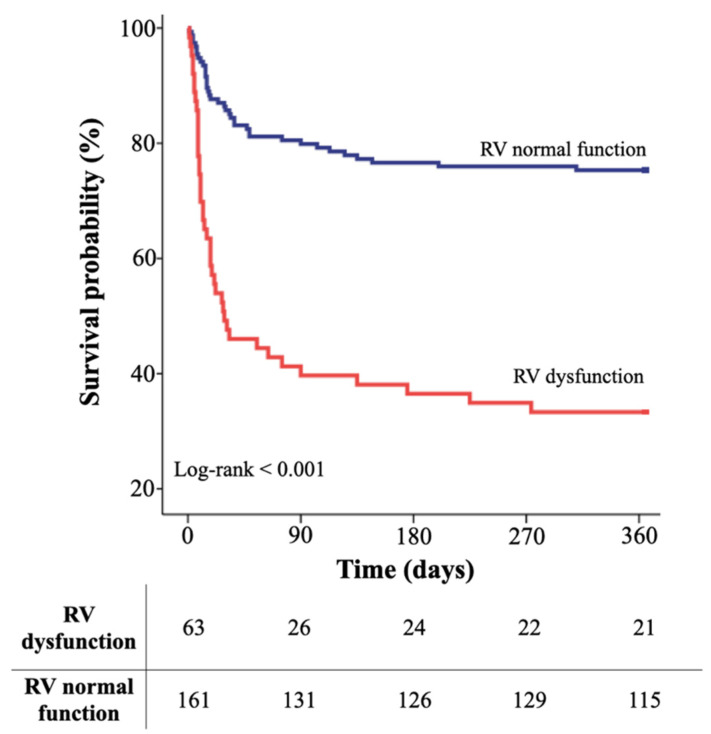
Kaplan–Meier curves for survival free from all-cause death in RV dysfunction (red line) vs. normal RV function (blue line) group.

The mortality cumulative incidence was significantly higher in patients with RV dysfunction at different follow-up times as compared to patients with normal RV function (30 days: 48.6 ± 5% vs. 14.3 ± 3%; 90 days: 60.3 ± 6% vs. 20.1 ± 3%; 365 days: 67.7 ± 6% vs. 24.7 ± 4%). Kaplan–Meier curves confirmed the significantly lower survival free from overall mortality in patients with vs. those without RV dysfunction (log-rank < 0.001; Figure 1).

After propensity score matching (1:1), 63 couples of patients with balanced baseline characteristics were found. The main baseline, clinical, and echocardiographic characteristics of the matched population are summarized in Table 1a, 1b. In the matched population, RV dysfunction was confirmed to be associated with 1-year mortality (67% vs. 37%; *p* < 0.001).

At univariable Cox regression analysis, age, male gender, hypertension, CAD, chronic heart failure, LVEF, TAPSE, ARDS, and pulmonary embolism were associated with 1-year mortality. At the multivariable analysis, RV dysfunction, along with LVEF and ARDS, emerged as an independent predictor of 1-year mortality (Table 3) and remained significantly associated after propensity score adjustment (C statistic = 0.870, *p* < 0.001; *p* = 0.672 for Hosmer–Lemeshow test).

## 4. Discussion

The main findings of this real-world multicenter study can be summarized as follows:

1. The long-term clinical outcome of hospitalized COVID-19 enrolled in this multicenter registry-based population was substantially unfavorable;
2. RV dysfunction is a common TTE finding in COVID-19 hospitalized patients;
3. TAPSE, as an easy-to-measure echocardiographic parameter of RV systolic function, was associated to higher incidence of long-term mortality, independently from age, comorbidities, and LVEF.

Data on the long-term outcome of patients infected by SARS-CoV-2 are still poor. However, the potential long-term cardiovascular sequelae are emerging as a global health problem [24]. Between November 2002 and August 2003, there were 8096 SARS cases globally with 900 deaths [25]. In a follow-up study enrolling SARS-CoV-1 survivors, 1-year follow-up identified abnormalities on chest X-ray in 28% of patients, the severity of lung damage was closely related to the extent of functional lung impairment, and the overall quality of life in SARS-CoV-1 survivors was worse than observed in an age-matched comparison cohort [26]. Since SARS-CoV-2 has been associated with high pathogenicity and invasiveness and has infected millions of people worldwide, it is important to report promptly the possible long-term sequelae of COVID-19 in order to plan adequate preventive and intervention strategies.

This multicenter registry-based study, although with a relatively limited sample size, provides timely information regarding long-term clinical outcome in COVID-19 patients. Our cohort of patients showed poor prognosis with a 1-year mortality rate of almost 40%, although it was partially driven by the high median age and the several comorbidities.

In this study, the prevalence of RV dysfunction is almost one-third of the entire population, confirming previous evidence; García-Cruz et al. and Mahmoud-Elsayed et al. reported RV in about 27% of COVID-19 patients [27,28]. The high prevalence of RV dysfunction could be explicated by the close heart–lung interactions, which have a pivotal role in COVID-19 clinical course. The distinctive hyper-inflammatory and pro-thrombotic state, the pulmonary micro-thrombosis, and the hypoxic pulmonary vasoconstriction secondary to extensive interstitial pneumonia play a synergistic role to start and sustain the spiral cascade leading to RV overload and failure; furthermore, COVID-19-related myocarditis, acute coronary syndromes, and arrhythmias could be other potential causes of acute RV dysfunction.

RV dysfunction could also affect the LV loading and LVEF by ventricular interdependence. The resulting reduction of the cardiac output, which impairs the blood supply to vital organs, can further aggravate the critical condition of the patient by exposing the overloaded RV to ischemia [29]. In COVID-19 patients, all this is amplified by the pulmonary microangiopathy with small vessel thrombosis, which explains the particularly high incidence of cardiorespiratory failure in critically ill COVID-19 patients [29]. The theoretical central role, as a long-term prognostic factor of RV dysfunction, was consistent with previous studies demonstrating the association between RV failure and in-hospital mortality [14,15,16].

Although in-hospital mortality is described as probable in patients with RV dysfunction at presentation, the novelty of our study is that these patients had a poor prognosis also up to 1 year.

Myocardial function and architecture have been extensively assessed using speckle tracking echocardiography and cardiac magnetic resonance; regardless of apparent normalization, the persistence of subtle ventricular systolic and diastolic abnormalities along with the development of microscopic fibrosis after myocardial oedema resolution has been demonstrated [30]. Puntmann et al. demonstrated a cardiovascular involvement, detected by standardized CMR irrespective of preexisting conditions, in patients who recovered from COVID-19 [31]. The most prevalent abnormality was myocardial inflammation (defined as abnormal native T1 and T2 measures), which was detected in 60% of the patients enrolled, followed by regional scar and pericardial enhancement [31]. Furthermore, in a multicenter study including 148 patients with COVID-19 and elevated serum troponin levels at admission at six hospitals, CMR showed myocardial damage in approximately half of the patients up to 3 months from discharge. Myocarditis-pattern injury was observed in 27% of cases, ischemic pattern was observed in 22% of patients, and non-specific LGE was observed in 5% of patients [32]. Combined ischemic and non-ischemic late gadolinium enhancement (LGE) was detected in 6% of cases. Interestingly, in patients with a myocarditis-like scar, ongoing active myocardial inflammation was described in a significant percentage of cases. We may hypothesize that COVID-19 patients with RV dysfunction also had a higher grade of persistent myocardial structural and metabolic impairment after discharge.

In the present analysis, patients with normal RV function had fewer comorbidities and lower D-dimer, troponin, and Pro-BNP serum levels compared with patients with RV systolic dysfunction. After matching, although Pro-BNP and D-dimer serum levels were balanced between groups, patients with RV dysfunction showed a higher risk of pulmonary embolism. D-dimer is usually abnormal in COVID-19, and it has a lower discriminative ability for this life-threatening complication than in the general population [12]. In this context, patients with RV disfunction assessed by TAPSE may have the highest risk of developing pulmonary embolism and its long-term sequelae. Indeed, within 2 years after a pulmonary embolism event, it has been reported a cumulative incidence of 0.1–9.1% of chronic thromboembolic pulmonary hypertension (CTPH), which is a distinct pulmonary vascular disease caused by the chronic obstruction of major pulmonary arteries [23] and associated with high mortality [33]. Such a mechanism might play a role also in COVID-19, and it may explain why patients with RV dysfunction at admission experienced worse 1-year outcome in a relevant percentage of cases.

First, RV dysfunction is highly probable in patients hospitalized with COVID-19 that should be considered as a life-threatening factor. Second, due to the high risk of mortality in the long term, COVID-19 patients complicated by RV dysfunction should be closely monitored after hospital discharge; whenever possible, they should be included in dedicated cardiovascular follow-up programs, particularly focused on RV assessment, that may be acknowledged as the cardiac chamber most affected in COVID-19 [34].

### Study Limitation

Our study is limited by the prospective design and the relatively small simple size. Second, the registry included only hospitalized COVID-19 patients; therefore, the results could not be generalized to the overall SARS-CoV-2 infected population, which is often characterized by an asymptomatic or poorly symptomatic forms. Third, we did not include indexes of diastolic dysfunction as well as other measures of global right ventricular function (e.g., RV fractional area change, DTI-derived S’-wave velocity), parameters from speckle tracking, or other advanced echocardiographic techniques. Indeed, the context of pandemic and the risk of infection for the operators limit the number of echocardiographic parameters routinely obtained in the enrolled patients and oriented to a clinical approach based on a limited standardized dataset quickly collected at bedside. Fourth, the pandemic and the urgent setting did not permit the assessment of inter- and intra-observer variability in echocardiographic measurements. Fifth, owing to the absence of TTE data before hospitalization and beyond 48 h after the admission, we cannot exclude the influence of pre-existent and/or late onset of LV and/or RV impairment. However, our aim was not to investigate the prognostic role of new-onset TTE abnormalities but to explore the association between echocardiographic findings at admission and long-term course in patients with COVID-19.

## 5. Conclusions

Data from our multicenter Italian registry demonstrated that RV dysfunction is a relatively common finding in hospitalized patients with COVID-19, and it is independently associated with an increased risk of 1-year mortality. Data from larger studies are warranted to confirm our preliminary findings.

## Figures and Tables

**Table 1:** (**a**) Clinical data (**b**) Echocardiographic data.

(a)						
Variables	Total (*n* = 224)	Normal RV Function (*n* = 161)	RV Dysfunction (*n* = 63)	*p*-Value	Matched Normal RV Function (*n* = 63)	*p*-Value
**Demographics**						
Age, years	69 ± 14	67 ± 14	75 ± 11	<0.001	71 ± 12	0.147
Male, %	140 (62)	102 (63)	38 (60)	0.673	45 (69)	0.262
**Past diagnosis**						
Hypertension, %	137 (61)	94 (58)	43 (68)	0.173	38 (60)	0.462
Diabetes, %	63 (28)	40 (25)	23 (36)	0.081	18 (28)	0.288
Dyslipidemia, %	60 (30)	38 (27)	22 (39)	0.113	24 (38)	0.729
CAD, %	35 (16)	18 (11)	17 (27)	0.003	12 (19)	0.289
Heart Failure, %	22 (10)	8 (5)	14 (22)	<0.001	8 (12)	0.159
COPD, %	45 (20)	21 (13)	24 (38)	<0.001	16 (25)	0.125
Stroke or TIA, %	17 (8)	10 (6)	7 (11)	0.213	3 (4)	0.187
CKD, %	45 (20)	20 (12)	25 (39)	<0.001	15 (24)	0.055
Cancer, %	27 (12)	21 (13)	6 (10)	0.467	9 (15)	0.409
**Cardiovascular drug at hospitalization**						
ACE-I or ARB, %	98 (44)	61 (38)	37 (58)	0.005	23 (37)	0.030
ß- blocker, %	59 (63)	38 (24)	21 (33)	0.137	20 (31)	0.618
Ca^++^ channel blocker, %	35 (16)	23 (14)	12 (19)	0.377	11 (18)	0.812
Antiplatelet, %	75 (33)	44 (27)	31 (49)	0.003	29 (46)	0.693
DAPT, %	12 (5)	3 (2)	9 (14)	<0.001	2 (3)	0.023
Anticoagulant, %	42 (19)	27 (17)	15 (24)	0.225	14 (22)	0.741
Statin, %	70 (31)	37 (23)	33 (52)	<0.001	21 (34)	0.030
**Symptoms**						
Fever, %	153 (68)	104 (65)	49 (77)	0.058	40 (63)	0.078
Dyspnoea, %	158 (70)	113 (70)	45 (71)	0.855	46 (73)	0.677
Chest discomfort, %	69 (31)	43 (27)	26 (41)	0.034	19 (29)	0.193
Cough, %	85 (38)	57 (35)	28 (44)	0.210	17 (27)	0.040
Sincope, %	21 (9)	14 (9)	7 (11)	0.577	6 (10)	0.520
**Serum Biomarkers**						
Troponin hs, n*99th percentile	1.6 (0.01–26.4)	0.1 (0.01–3.36)	25 (1–146)	<0.001	0.1 (0.01–25)	0.022
D-dimer, ng/mL	376 (34–650)	204 (10–356)	887 (133–2158)	0.008	655 (20–2254)	0.232
Pro-BNP, pg/mL	2007 (300–8941)	895 (288–6654)	2625 (482–11,775)	0.104	2750 (731–12,550)	0.388
**COVID-19 therapies**						
Antibiotics, %	165 (74)	117 (73)	48 (76)	0.591	47 (75)	0.955
Glucocorticoids, %	100 (45)	66 (41)	34 (54)	0.079	28 (44)	0.343
UFH or LMWH, %	181 (82)	132 (83)	49 (78)	0.364	53 (84)	0.364
**(b)**						
**Variables**	**Total** **(*n* = 224)**	**Normal RV Function** **(*n* = 161)**	**RV Dysfunction** **(*n* = 63)**	***p*-Value**	**Matched Normal RV Function** **(*n* = 63)**	***p*-Value**
LVEF	55 (50–59)	55 (53–60)	50 (43–55)	<0.001	53 (50–57)	0.104
LVEDV	103 (89–120)	104 (89–121)	99 (88–118)	0.282	105 (92–120)	0.095
LVESV	47 (39–58)	46 (38–56)	48 (41–60)	0.613	50 (41–60)	0.351
TAPSE, mm	21 (18–23)	22 (20–24)	16 (14–17)	<0.001	22 (21–24)	<0.001
PAPS, mmHg	33 (30–41)	30 (29–38)	43 (37–49)	<0.001	31 (28–38)	<0.001
Pulmonary Hypertension, %	108 (48)	55 (32)	53 (85)	<0.001	26 (41)	<0.001

CAD, coronary artery disease; COPD, chronic obstructive pulmonary disease; TIA, transient ischemic attack; CKD, chronic kidney disease; ACE-I, angiotensin-converting enzyme inhibitor; ARB, angiotensin receptor blocker; DAPT, dual antiplatelet therapy; UFH, unfractionated heparin; LMWH, low molecular weight heparin. LVEF, left ventricle ejection fraction; LVEDV, left ventricular end diastolic volume; LVESV, left ventricular end systolic volume; TAPSE, tricuspid annular plane systolic excursion.

**Table 2:** Clinical outcome of study population.

Variables	Total (*n* = 224)	Normal RV Function (*n* = 161)	RV Dysfunction (*n* = 63)	*p*-Value	Matched Normal RV Function (*n* = 63)	*p*-Value *
Mortality- 1 year	87 (39)	45 (28)	42 (67)	<0.001	23 (37)	<0.001
In-hospital mortality	68 (30)	29 (18)	39 (62)	<0.001	18 (29)	<0.001
Cardiac injury	69 (31)	33 (20)	36 (57)	<0.001	17 (27)	0.001
ARDS	107 (48)	72 (45)	35 (56)	0.144	30 (49)	0.514
Pulmonary embolism	32 (14)	14 (9)	18 (29)	<0.001	4 (6)	<0.001

ARDS, acute respiratory distress syndrome. * Matched normal RV vs. RV disfunction.

**Table 3:** Univariable and multivariable analysis for of the risk of 1-year mortality.

Variables	Univariable HR (95% CI)	*p*	Multivariable HR (95% CI)	*p*
LVEF (%)	0.93 (0.91–0.95)	<0.001	0.97 (0.94–0.99)	0.043
TAPSE (mm)	0.84 (0.80–0.88)	<0.001	0.87 (0.81–0.93)	<0.001
Age (per year)	1.02 (1.01–1.06)	0.008	-	-
ARDS	6.34 (3.53–11.38)	<0.001	5.88 (3.17–10.91)	<0.001
Pulmonary embolism	1.94 (1.12–3.33)	0.017	-	-
Male gender	1.77 (1.06–2.96)	0.029	-	-
Hypertension	1.78 (1.07–2.94)	0.026	-	-
CAD	1.74 (1.00–3.02)	0.050	-	-
Chronic heart failure	2.05 (1.13–3.74)	0.018	-	-

LVEF, left ventricular ejection fraction; TAPSE, tricuspid annular plane systolic excursion; ARDS, acute respiratory distress syndrome; CAD, coronary artery disease.

## Data Availability

The data that support the findings of this study are available from the corresponding author, G.P., upon reasonable request.

## References

[1] Silverio A., DI Maio M., Ciccarelli M., Carrizzo A., Vecchione C., Galasso G. (2020). Timing of national lockdown and mortality in COVID-19: The Italian experience. Int. J. Infect. Dis..

[2] Richardson S., Hirsch J.S., Narasimhan M., Crawford J.M., McGinn T., Davidson K.W., Northwell COVID-19 Research Consortium (2020). Presenting Characteristics, Comorbidities, and Outcomes Among 5700 Patients Hospitalized with COVID-19 in the New York City Area. JAMA.

[3] Russo V., Bottino R., D’Andrea A., Silverio A., Di Maio M., Golino P., Nigro G., Valsecchi O., Attena E., Canonico M.E. (2021). Chronic Oral Anticoagulation and Clinical Outcome in Hospitalized COVID-19 Patients. Cardiovasc. Drugs Ther..

[4] Silverio A., Di Maio M., Citro R., Esposito L., Iuliano G., Bellino M., Baldi C., De Luca G., Ciccarelli M., Vecchione C. (2021). Cardiovascular risk factors and mortality in hospitalized patients with COVID-19: Systematic review and meta-analysis of 45 studies and 18,300 patients. BMC Cardiovasc. Disord..

[5] Zu W., McGoogan J.M. (2020). Characteristics of and important lessons from the coronavirus disease 2019 (COVID-19) outbreak in China: Summary of a report of 72314 cases from the Chinese Center for Disease Control and Prevention. JAMA.

[6] Petrilli C.M., Jones S.A., Yang J., Rajagopalan H., O’Donnell L., Chernyak Y., Tobin K.A., Cerfolio R.J., Francois F., Horwitz L.I. (2020). Factors associated with hospital admission and critical illness among 5279 people with coronavirus disease 2019 in New York City: Prospective cohort study. BMJ.

[7] Madjid M., Safavi-Naeini P., Solomon S.D., Vardeny O. (2020). Potential Effects of Coronaviruses on the Cardiovascular System: A Review. JAMA Cardiol..

[8] Russo V., Silverio A., Scudiero F., Di Micco P., Di Maio M. (2021). Pre-admission atrial fibrillation in COVID-19 patients: Prevalence and clinical impact. Eur. J. Intern. Med..

[9] Scudiero F., Pitì A., Keim R., Parodi G. (2021). Acute pulmonary embolism in COVID-19 patient: A case report of free-floating right heart thrombus successfully treated with fibrinolysis. Eur. Hear. J. Case Rep..

[10] Park J.F., Banerjee S., Umar S. (2020). In the eye of the storm: The right ventricle in COVID-19. Pulm. Circ..

[11] Russo V., Di Maio M., Mottola F.F., Pagnano G., Attena E., Verde N., Di Micco P., Silverio A., Scudiero F., Nunziata L. (2020). Clinical characteristics and prognosis of hospitalized COVID-19 patients with incident sustained tachyarrhythmias: A multicenter observational study. Eur. J. Clin. Investig..

[12] Scudiero F., Silverio A., Di Maio M., Russo V., Citro R., Personeni D., Cafro A., D’Andrea A., Attena E., Pezzullo S. (2021). Pulmonary embolism in COVID-19 patients: Prevalence, predictors and clinical outcome. Thromb. Res..

[13] Pagnesi M., Baldetti L., Beneduce A., Calvo F., Gramegna M., Pazzanese V., Ingallina G., Napolano A., Finazzi R., Ruggeri A. (2020). Pulmonary hypertension and right ventricular involvement in hospitalised patients with COVID-19. Heart.

[14] Li Y., Li H., Zhu S., Xie Y., Wang B., He L., Zhang D., Zhang Y., Yuan H., Wu C. (2020). Prognostic Value of Right Ventricular Longitudinal Strain in Patients With COVID-19. JACC Cardiovasc. Imaging.

[15] Keskin M., Uzun A.O., Hayıroğlu M.İ., Kaya A., Çınar T., Kozan Ö. (2018). The association of right ventricular dysfunction with in-hospital and 1-year outcomes in anterior myocardial infarction. Int. J. Cardiovasc. Imaging.

[16] Iglesias-Garriz I., Olalla-Gómez C., Garrote C., López-Benito M., Martín J., Alonso D., A Rodríguez M. (2012). Contribution of right ventricular dysfunction to heart failure mortality: A meta-analysis. Rev. Cardiovasc. Med..

[17] WHO Clinical Management of Severe Acute Respiratory Infection When Novel Coronavirus (2019-nCoV) Infection is Suspected: Interim Guidance. 28 January 2020. https://www.who.int/docs/defaultsource/coronaviruse/clinical-management-of-novel-cov.pdf.

[18] Silverio A., Di Maio M., Scudiero F., Russo V., Esposito L., Attena E., Pezzullo S., Parodi G., D’Andrea A., Damato A. (2021). Clinical conditions and echocardiographic parameters associated with mortality in COVID-19. Eur. J. Clin. Investig..

[19] Ranieri V.M., Rubenfeld G.D., Thompson B.T., Ferguson N.D., Caldwell E., Fan E., Camporota L., Slutsky A.S., ARDS Definition Task Force (2012). Acute respiratory distress syndrome: The Berlin Definition. JAMA.

[20] Thygesen K., Alpert J.S., Jaffe A.S., Chaitman B.R., Bax J.J., Morrow D.A., White H.D., ESC Scientific Document Group (2019). Fourth universal definition of myocardial infarction. Eur. Heart J..

[21] Jain S.S., Liu Q., Raikhelkar J., Fried J., Elias P., Poterucha T.J., DeFilippis E.M., Rosenblum H., Wang E.Y., Redfors B. (2020). Indications for and Findings on Transthoracic Echocardiography in COVID-19. J. Am. Soc. Echocardiogr..

[22] Lang R.M., Badano L.P., Mor-Avi V., Afilalo J., Armstrong A., Ernande L., Flachskampf F.A., Foster E., Goldstein S.A., Kuznetsova T. (2015). Recommendations for Cardiac Chamber Quantification by Echocardiography in Adults: An Update from the American Society of Echocardiography and the European Association of Cardiovascular Imaging. J. Am. Soc. Echocardiogr..

[23] Galiè N., Humbert M., Vachiéry J.-L., Gibbs S., Lang I.M., Kaminski K.A., Simonneau G., Peacock A., Noordegraaf A.V., Beghetti M. (2016). 2015 ESC/ERS Guidelines for the diagnosis and treatment of pulmonary hypertension: The Joint Task Force for the Diagnosis and Treatment of Pulmonary Hypertension of the European Society of Cardiology (ESC) and the European Respiratory Society (ERS): Endorsed by: Association for European Paediatric and Congenital Cardiology (AEPC), International Society for Heart and Lung Transplantation (ISHLT). Eur. Heart J..

[24] Wang F., Kream R.M., Stefano G.B. (2020). Long-Term Respiratory and Neurological Sequelae of COVID-19. Med. Sci. Monit..

[25] Ngai J.C., Ko F.W., Ng S.S., To K.-W., Tong M., Hui D.S. (2010). The long-term impact of severe acute respiratory syndrome on pulmonary function, exercise capacity and health status. Respirology.

[26] Hui D.S., Wong K.T., Ko F.W.S., Tam L.-S., Chan D.P., Woo J., Sung J.J.Y. (2005). The 1-Year Impact of Severe Acute Respiratory Syndrome on Pulmonary Function, Exercise Capacity, and Quality of Life in a Cohort of Survivors. Chest.

[27] García-Cruz E., Manzur-Sandoval D., Rascón-Sabido R., Gopar-Nieto R., Barajas-Campos R.L., Jordán-Ríos A., Martínez D.S., Jiménez-Rodríguez G.M., Murillo-Ochoa A.L., Díaz-Méndez A. (2020). Critical care ultrasonography during COVID-19 pandemic: The ORACLE protocol. Echocardiography.

[28] Mahmoud-Elsayed H.M., Moody W.E., Bradlow W.M., Khan-Kheil A.M., Hudsmith L.E., Steeds R.P. (2020). Echocardiographic Findings in COVID-19 Pneumonia. Can. J. Cardiol..

[29] Dandel M. (2021). Heart–lung interactions in COVID-19: Prognostic impact and usefulness of bedside echocardiography for monitoring of the right ventricle involvement. Hear. Fail. Rev..

[30] Schwarz K., Ahearn T., Srinivasan J., Neil C.J., Scally C., Rudd A., Jagpal B., Frenneaux M.P., Pislaru C., Horowitz J.D. (2017). Alterations in Cardiac Deformation, Timing of Contraction and Relaxation, and Early Myocardial Fibrosis Accompany the Apparent Recovery of Acute Stress-Induced (Takotsubo) Cardiomyopathy: An End to the Concept of Transience. J. Am. Soc. Echocardiogr..

[31] Puntmann V.O., Carerj M.L., Wieters I., Fahim M., Arendt C., Hoffmann J., Shchendrygina A., Escher F., Vasa-Nicotera M., Zeiher A.M. (2020). Outcomes of Cardiovascular Magnetic Resonance Imaging in Patients Recently Recovered From Coronavirus Disease 2019 (COVID-19). JAMA Cardiol..

[32] Kotecha T., Knight D.S., Razvi Y., Kumar K., Vimalesvaran K., Thornton G., Patel R., Chacko L., Brown J.T., Coyle C. (2021). Patterns of myocardial injury in recovered troponin-positive COVID-19 patients assessed by cardiovascular magnetic resonance. Eur. Hear. J..

[33] Lang I.M. (2004). Chronic Thromboembolic Pulmonary Hypertension—Not So Rare after All. N. Engl. J. Med..

[34] Szekely Y., Lichter Y., Taieb P., Banai A., Hochstadt A., Merdler I., Oz A.G., Rothschild E., Baruch G., Peri Y. (2020). Spectrum of Cardiac Manifestations in COVID-19: A Systematic Echocardiographic Study. Circulation.

